# A large-scale genetic screen for mutants with altered salicylic acid accumulation in Arabidopsis

**DOI:** 10.3389/fpls.2014.00763

**Published:** 2015-01-07

**Authors:** Yezhang Ding, Danjela Shaholli, Zhonglin Mou

**Affiliations:** Department of Microbiology and Cell Science, University of FloridaGainesville, FL, USA

**Keywords:** salicylic acid, genetic screen, *NPR1*, *Arabidopsis thaliana*, disease resistance, *sln* mutant, *isn* mutant

## Abstract

Salicylic acid (SA) is a key defense signal molecule against biotrophic and hemibiotrophic pathogens in plants, but how SA is synthesized in plant cells still remains elusive. Identification of new components involved in pathogen-induced SA accumulation would help address this question. To this end, we performed a large-scale genetic screen for mutants with altered SA accumulation during pathogen infection in Arabidopsis using a bacterial biosensor *Acinetobacter* sp. ADPWH_*lux*-based SA quantification method. A total of 35,000 M_2_ plants in the *npr1-3* mutant background have been individually analyzed for the bacterial pathogen *Pseudomonas syringae* pv. *maculicola* (*Psm*) ES4326-induced SA accumulation. Among the mutants isolated, 19 had SA levels lower than *npr1* (*sln*) and two exhibited increased SA accumulation in *npr1* (*isn*). Complementation tests revealed that seven of the *sln* mutants are new alleles of *eds5*/*sid1*, two are *sid2*/*eds16* alleles, one is allelic to *pad4*, and the remaining seven *sln* and two *isn* mutants are new non-allelic SA accumulation mutants. Interestingly, a large group of mutants (in the *npr1-3* background), in which *Psm* ES4326-induced SA levels were similar to those in the wild-type Columbia plants, were identified, suggesting that the signaling network fine-tuning pathogen-induced SA accumulation is complex. We further characterized the *sln1* single mutant and found that *Psm* ES4326-induced defense responses were compromised in this mutant. These defense response defects could be rescued by exogenous SA, suggesting that *SLN1* functions upstream of SA. The *sln1* mutation was mapped to a region on the north arm of chromosome I, which contains no known genes regulating pathogen-induced SA accumulation, indicating that *SLN1* likely encodes a new regulator of SA biosynthesis. Thus, the new *sln* and *isn* mutants identified in this genetic screen are valuable for dissecting the molecular mechanisms underlying pathogen-induced SA accumulation in plants.

## Introduction

As sessile organisms, plants are under constant attack from diverse microbes including bacteria, fungi, oomycetes, and viruses. To ward off pathogens, plants activate their immune system to mount multiple defense responses, which are similar to animal innate immunity (Jones and Dangl, 2006). Recognition of pathogen-associated molecular patterns (PAMPs) by pattern recognition receptors results in PAMP-triggered immunity (PTI). To achieve successful colonization, adapted pathogens can deliver effector molecules directly into the plant cells to suppress PTI, resulting in effector-triggered susceptibility (ETS) (Jones and Dangl, 2006). On the other hand, plants have evolved resistance (R) proteins to detect the presence of certain pathogen effector molecules, inducing effector-triggered immunity (ETI). Activation of PTI or ETI leads to generation of mobile signals, which induce a long-lasting broad-spectrum immune response known as systemic acquired resistance (SAR) (Durrant and Dong, 2004).

The phytohormone salicylic acid (SA) plays an essential role in these defense response pathways (Vlot et al., 2009). Exogenous application of SA or its analogs induces expression of defense genes including *PR* (*pathogenesis-related*) genes and disease resistance (White, 1979; Dong, 2004), whereas transgenic plants carrying the bacterial *NahG* gene, which encodes an SA hydroxylase, are hypersusceptible to pathogen infection and fail to develop SAR (Gaffney et al., 1993; Delaney et al., 1994; Lawton et al., 1995). Furthermore, Arabidopsis mutants with impaired SA biosynthesis during pathogen infection, such as *sid2* (*salicylic acid induction-deficient2*) (Nawrath and Métraux, 1999; Wildermuth et al., 2001), *eds5* (*enhanced disease susceptibility5*) (Nawrath and Métraux, 1999; Nawrath et al., 2002), and *pad4* (*phytoalexin deficient4*) (Zhou et al., 1998; Jirage et al., 1999), show compromised defense responses. In contrast, mutants with elevated levels of SA, such as *acd* (*accelerated cell death*) (Greenberg et al., 1994; Rate et al., 1999), *cpr* (*constitutive expressor of PR genes*) (Bowling et al., 1997; Clarke et al., 1998), and *ssi* (*suppressor of salicylate insensitivity of npr1-5*) (Shah et al., 1999, 2001), display constitutive expression of *PR* genes and SAR.

Previous research has revealed that plants mainly utilize two distinct enzymatic pathways to synthesize SA, the phenylalanine ammonia-lyase (PAL) pathway and the isochorismate (IC) pathway (Vlot et al., 2009; Dempsey et al., 2011). Both pathways require the primary metabolite chorismate, which is derived from the shikimate pathway. Earlier studies using isotope feeding suggested that SA is synthesized from phenylalanine via either benzoate intermediates or coumaric acid catalyzed by a series of enzymes including PAL, benzoic acid 2-hydroxylase, and other unknown enzymes (León et al., 1995; Dempsey et al., 2011). SA can also be synthesized through isochorismate catalyzed by isochorismate synthase (ICS) and isochorismate pyruvate lyase (IPL). Two ICS enzymes, ICS1 and ICS2, exist in Arabidopsis, and ICS1 has been shown to play a major role in SA biosynthesis (Garcion et al., 2008). Intriguingly, no plant genes encoding IPL have been identified. In comparison to the PAL pathway, the IC pathway plays a more important role in synthesis of both basal and induced SA in Arabidopsis (Mauch-Mani and Slusarenko, 1996; Garcion et al., 2008). However, neither pathway has been fully defined so far.

Nawrath and Métraux (1999) conducted a forward genetic screen in Arabidopsis for mutants with altered levels of total SA after infection with the bacterial pathogen *Pseudomonas syringae* pv. *tomato* (*Pst*) DC3000 carrying the avirulence gene *avrRpm1*. Two mutants, *sid1* and *sid2*, were identified, which did not accumulate SA during the infection (Nawrath and Métraux, 1999). The *sid1* and *sid2* mutants were shown to be allelic to *eds5* and *eds16*, respectively, which were identified in another genetic screen for enhanced disease susceptibility (Rogers and Ausubel, 1997; Nawrath and Métraux, 1999). *EDS5*/*SID1* encodes a chloroplast MATE (multidrug and toxin extrusion) transporter (Nawrath et al., 2002), and *SID2*/*EDS16* encodes an SA biosynthetic enzyme ICS1 (Wildermuth et al., 2001). In this screen, an HPLC (high performance liquid chromatography)-based method was used to quantify SA levels in pathogen-infected leaf tissues from about 4500 individual M_2_ plants. Obviously, the genetic screen did not reach saturation.

The HPLC-based method used by Nawrath and Métraux (1999) is extremely costly and time-consuming, which would not be practical for a large-scale genetic screen. Recently, an SA biosensor, named *Acinetobacter* sp. ADPWH_*lux*, was developed (Huang et al., 2005). This bacterial strain was derived from *Acinetobacter* sp. ADP1 and contains a chromosomal integration of an SA-inducible *lux*-*CDABE* operon, which encodes a luciferase (LuxA and LuxB) and the enzymes that produce its substrate (LuxC, LuxD, and LuxE). In the presence of SA, methylsalicylic acid, and acetylsalicylic acid, the operon is activated, resulting in emission of 490-nm light (Huang et al., 2005). Measurement of SA from tobacco mosaic virus-infected tobacco leaves with the biosensor and gas chromatography/mass spectrometry (GC/MS) yielded similar results, demonstrating that this strain is suitable for quantification of SA in plants (Huang et al., 2006). DeFraia et al. developed an improved methodology for *Acinetobacter* sp. ADPWH_*lux*-based SA quantification for both free SA and SA *O*-β-glucoside (SAG) in crude plant extracts (Defraia et al., 2008). Based on this, Marek et al. (2010) established a further simplified protocol for estimation of free SA levels in crude plant extracts in a high-throughput format (Marek et al., 2010). The efficacy and effectiveness of the newly developed SA biosensor-based method were confirmed by HPLC and verified in a small-scale mutant screen.

To better understand SA biology, we conducted a large-scale forward genetic screen aimed at isolating more Arabidopsis mutants with altered SA accumulation upon pathogen infection. We expected that mutants accumulating significantly altered levels of SA during pathogen infection will help study how SA is synthesized in plant cells and uncover important regulators of plant immunity. This screen allowed us to identify nine new mutants with significantly altered levels of pathogen-induced SA in the *npr1-3* genetic background. Among them, seven produced SA levels lower than *npr1* (*sln*) and two displayed increased SA accumulation in *npr1* (*isn*). Enhanced disease resistance tests demonstrated that the seven new *sln npr1-3* mutants are more susceptible to bacterial pathogen infection, while both *isn npr1-3* mutants are more resistant than *npr1-3*. We further characterized the *sln1* single mutant and found that the *sln1* mutation compromised the bacterial pathogen *P. syringae* pv. *maculicola* (*Psm*) ES4326-induced defense responses. Moreover, exogenous SA induced both *PR* gene expression and disease resistance in *sln1*, indicating that *SLN1* functions upstream of SA. Finally, the *sln1* mutation was mapped to a region on the north arm of chromosome I, which contains no known genes involved in regulating pathogen-induced SA accumulation, suggesting that *SLN1* encodes a new SA pathway component.

## Materials and methods

### Plant materials and growth conditions

The wild type used was the *Arabidopsis thaliana* (L.) Heynh. Columbia (Col-0) ecotype, and the mutant alleles used were *npr1-3* (Glazebrook et al., 1996), *npr1*-L (GT_5_89558), *eds5-1* (Nawrath et al., 2002), *sid2-1* (Nawrath and Métraux, 1999; Wildermuth et al., 2001), *pad4-1* (Glazebrook et al., 1996; Jirage et al., 1999), *eps1-1* (Zheng et al., 2009), and *pbs3-1* (Nobuta et al., 2007). The *eds5-1 npr1-3*, *sid2-1 npr1-3*, and *pad4-1 npr1-3* double mutants were created by crossing *npr1-3* with *eds5-1*, *sid2-1*, and *pad4-1*, respectively. Homozygous plants were identified by genotyping (Tables S1 and S2). Arabidopsis seeds were sown on autoclaved soil (Sunshine MVP, Sun Gro Horticulture, http://www.sungro.com) and cold-treated at 4°C for 3 days. Plants were grown at approximately 22°C under a 16-h light/8-h dark regime.

### Pathogen infection

The bacterial strains *Psm* ES4326 and *Pst* DC3000/*avrRpt2* were grown overnight in liquid King's B medium. Bacterial cells were collected by centrifugation and diluted in 10 mM MgCl_2_. Inoculation of plants was performed by pressure infiltration with a 1 mL needleless syringe (Clarke et al., 1998). For SA measurement, *Psm* ES4326 and *Pst* DC3000/*avrRpt2* suspensions with an OD_600_ of 0.001 were used for inoculation. The susceptibility phenotype was tested using a low-titer inoculum (OD_600_ = 0.0001) of *Psm* ES4326. *In planta* growth of *Psm* ES4326 was assayed 3 days after inoculation as previously described (Clarke et al., 1998). For SA-induced resistance assay, SA-treated plants were inoculated with a *Psm* ES4326 suspension (OD_600_ = 0.001) and the bacterial growth was determined 3 days post-inoculation.

### SA measurement

Free SA measurement using the SA biosensor was conducted as described by Marek et al. (2010). SA measurement with HPLC was performed as described by Verberne et al. (2002).

### RNA extraction and quantitative PCR

RNA extraction was carried out as described previously (Cao et al., 1997). For reverse transcription (RT), ~10 μg of total RNA was treated with DNase I (Ambion) at 37°C for 30 min for digestion of contaminating DNA. After inactivation of the DNase, ~2 μg of total RNA was used as a template for first-strand cDNA synthesis using the M-MLV Reverse Transcriptase first-strand synthesis system (Promega). The resulting cDNA products were diluted 20-fold with autoclaved distilled water, and 2.5 μL of the diluted solution was used for quantitative PCR (qPCR). qPCR was performed in an Mx3005P qPCR system (Stratagene). All qPCR reactions were performed with a 12.5 μL reaction volume using the SYBR Green protocol under the following conditions: denaturation program (95°C for 10 min), amplification and quantification program repeated for 40 cycles (95°C for 30 s, 55°C for 1 min, 72°C for 1 min), and melting curve program (95°C for 1 min, 55°C for 30 s, and 95°C for 30 s). The primers used for qPCR in this study are listed in Table S2.

### Statistical methods

Statistical analyses were performed with Prism 5 (GraphPad Software, Inc., La Jolla, CA). One-Way analysis of variance (ANOVA) was used to determine statistical significance among genotypes or treatments. In addition, two-way analysis of variance was used to examine the effects of genotypes, treatments, and the interaction of these two factors on disease resistance. Post-hoc comparison was performed using Fisher's least significant difference LSD test and represented by different letters. Alternatively, statistical analyses were performed using Student's *t*-test for comparison of two data sets (Assuming Unequal Variances).

### Accession number

The locus numbers for the genes discussed in this study are as follows: *NPR1* (At1g64280), *EDS5* (At4g39030), *ICS1* (At1g74710), *PAD4* (At3g52430), *EPS1* (At5g67160), *PBS3* (At5g13320), *PR1* (At2g14610), *PR2* (At3g57260), *PR5* (At1g75040), *UBQ5* (At3g62250).

## Results

### Isolation of SA accumulation mutants

**Figure 1:**
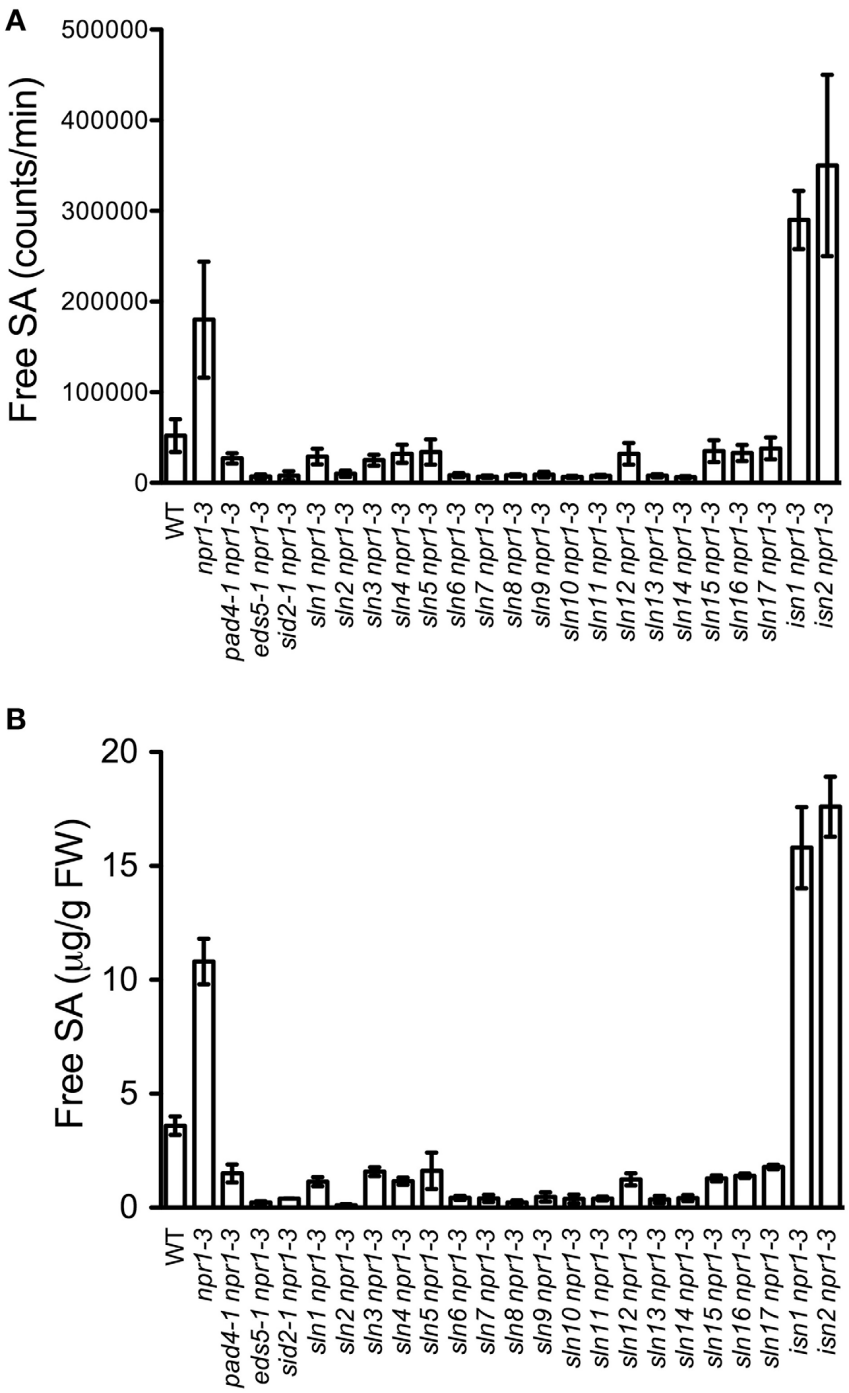
**Pathogen-induced free SA levels in the SA accumulation mutants. (A)** Luminescence from crude extracts of *Psm* ES4326-infected wild-type, *npr1-3, pad4-1 npr1-3, eds5-1 npr1-3, sid2-1 npr1-3*, and 19 putative mutant leaf tissues measured with the SA biosensor. **(B)** Free SA levels in *Psm* ES4326-infected wild-type, *npr1-3, pad4-1 npr1-3, eds5-1 npr1-3*, *sid2-1 npr1-3*, and 19 putative mutant plants detected by the HPLC-based method. Values are the mean of eight **(A)** or three **(B)** samples with standard deviation (SD). The experiments were repeated three times with similar results.

In order to identify new components involved in pathogen-induced SA accumulation, we took advantage of the SA biosensor-based method to screen for mutants with altered levels of pathogen-induced SA in Arabidopsis. Approximately 35,000 M_2_ plants from an ethyl methanesulfonate-mutagenized population (20 pools, each from ~500 M_1_ plants) in the *npr1-3* mutant background were individually analyzed for free SA levels after infection with the bacterial pathogen *Psm* ES4326. The *npr1-3* mutant was used as the starting material for the genetic screen, because it accumulates significantly higher levels of SA than wild type upon bacterial pathogen infection (Figures 1A,B; Cao et al., 1997; Ryals et al., 1997; Shah et al., 1997; Zhang et al., 2010). Plants that accumulated significantly higher or lower levels of pathogen-induced SA than *npr1-3* were considered to be putative SA accumulation mutants. Approximately 350 such mutants were identified in the primary screen. To confirm these putative mutants, eight plants of each mutant line were tested for *Psm* ES4326-induced SA accumulation using the SA biosensor in the M_3_ generation (Marek et al., 2010). Nineteen mutants with drastically altered levels of pathogen-induced SA, including 17 *sln npr1-3* and two *isn npr1-3* mutants, were chosen for further analysis (Figure 1A). SA levels accumulated in the remaining mutants were significantly lower than those in *npr1-3*, but slightly higher than those in the wild-type plants (data not shown). Contamination from other mutants in the lab was excluded by checking the mutant plants under ultraviolet (UV) illumination, since the *npr1-3* mutant carries a *fuhl-2* allele, which lacks sinapoyl malate in the leaf epidermis and appears red under UV light (Chapple et al., 1992; Glazebrook et al., 1996). In addition, the presence of the *npr1-3* mutation in the identified mutants was confirmed with a derived cleaved amplification polymorphism sequence (dCAPS) marker (Table S1).

To confirm that the 19 mutants accumulate altered levels of SA after pathogen infection, we measured free SA levels accumulated in these mutants after *Psm* ES4326 infection using HPLC. Similarly to the results obtained using the SA biosensor, upon *Psm* ES4326 infection, the 17 *sln npr1-3* mutants accumulated dramatically lower levels of free SA and the two *isn npr1-3* mutants produced higher levels of free SA than the *npr-3* mutant (Figure 1B). These results suggest that the *sln* mutations may reside in genes that are required for pathogen-induced SA biosynthesis, whereas the *isn* mutations may be located in suppressors of SA accumulation.

### Pathogen resistance of the SA accumulation mutants

**Figure 2:**
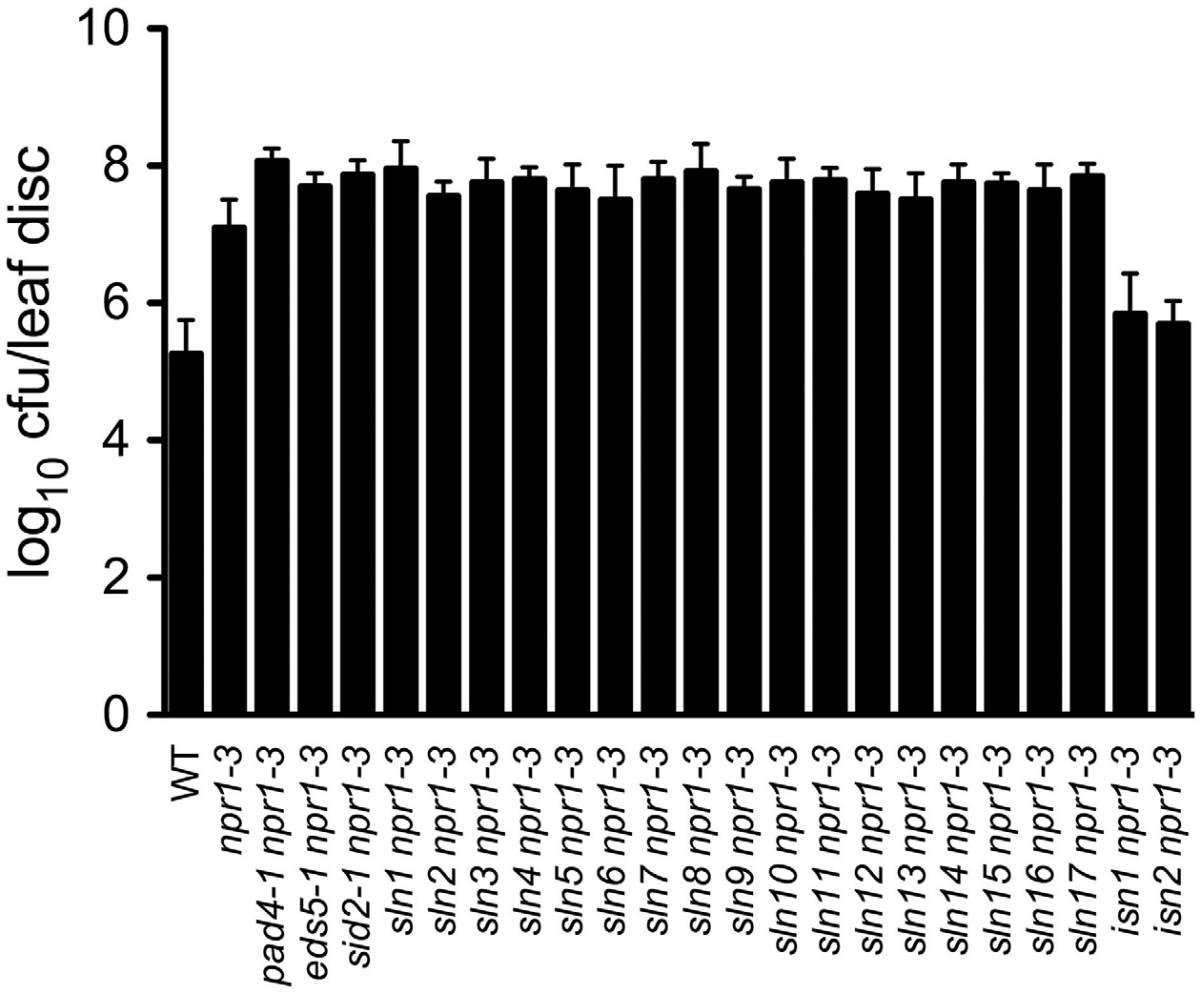
**Pathogen growth in the SA accumulation mutants**. Leaves of 4-week-old plants were inoculated with a *Psm* ES4326 suspension (OD_600_ = 0.0001). The *in planta* bacterial titers were determined 3 days post-inoculation. Data represent the mean of eight independent samples with SD. cfu, colony-forming units. The experiment was repeated three times with similar results.

SA accumulation is generally associated with resistance to biotrophic and hemibiotrophic bacterial pathogens (An and Mou, 2011). To investigate whether susceptibility or resistance to bacterial pathogens in the 19 SA accumulation mutants described above is also affected, we inoculated 4-week-old plants with a low-titer inoculum (OD_600_ = 0.0001) of the virulent bacterial pathogen *Psm* ES4326. Interestingly, all *sln npr1-3* mutants developed enhanced disease symptoms (data not shown) and supported more bacterial growth (2- to 7-fold) compared with the *npr1-3* mutant (Figure 2), suggesting that the *SLN* genes are required for resistance to the bacterial pathogen. In contrast, the two *isn npr1-3* mutants supported less *Psm* ES4326 growth than *npr1-3*, although the bacteria still grew to a slightly higher titer in the *isn npr1-3* mutants than in the wild-type plants (Figure 2), indicating that the increased levels of SA in the *isn npr1-3* mutants may activate NPR1-independent disease resistance.

### Allelism test

Analyses of the F_1_ plants from crosses between the 19 SA accumulation mutants and *npr1-3* indicated that all *sln* and *isn* mutations are recessive. Several recessive mutations, including *eds5* (Nawrath and Métraux, 1999; Nawrath et al., 2002), *sid2* (Nawrath and Métraux, 1999; Wildermuth et al., 2001), *pad4* (Glazebrook et al., 1996; Zhou et al., 1998; Jirage et al., 1999), *eds1* (Parker et al., 1996; Falk et al., 1999), *eps1* (Zheng et al., 2009), and *pbs3*/*win3*/*gdg1* (Jagadeeswaran et al., 2007; Lee et al., 2007; Nobuta et al., 2007), have been shown to compromise pathogen-induced SA accumulation. We reasoned that the *sln* mutants are unlikely alleles of *eps1*, *pbs3*, and *eds1*, since no difference in pathogen-induced free SA levels was detected between *eps1-1* or *pbs3-1* and the wild type using the SA biosensor (Figure S1), and two *EDS1* genes are present in the Arabidopsis ecotype Col-0 (Feys et al., 2005). We therefore tested for allelism between the *sln* mutants and *eds5*, *sid2*, or *pad4*. Pathogen-induced SA levels in F_1_ plants were measured using the SA biosensor and compared with those in their parents. These allelism tests revealed that seven *sln* mutants are alleles of *eds5*, two are *sid2* alleles, and one is allelic to *pad4* (Table 1).

**Table 1:** **Mutants identified in this genetic screen**.

**Gene/locus**	**Alleles/new mutants**
*SID1/EDS5*	*sln2*, *sln6*, *sln8*, *sln9*, *sln11*, *sln13*, *sln14*
*SID2/EDS16*	*sln7*, *sln10*
*PAD4*	*sln12*
*SLN1*	*sln1*
*SLN3*	*sln3*
*SLN4*	*sln4*
*SLN5*	*sln5*
*SLN15*	*sln15*
*SLN16*	*sln16*
*SLN17*	*sln17*
*ISN1*	*isn1*
*ISN2*	*isn2*

We also performed complementation tests for allelism among the remaining seven *sln* mutants. They were crossed to each other and the resulting F_1_ plants were tested for the ability to accumulate SA after *Psm* ES4326 infection using the SA biosensor. We found that the *sln* mutations complemented each other, suggesting that they are located in different genes required for pathogen-induced SA accumulation (Table 1). Moreover, complementation test indicated that the two *isn* mutations reside in two different genes, which are likely involved in suppressing pathogen-induced SA accumulation (Table 1).

### Characterization of the *sln1 npr1-3* mutant

**Figure 3:**
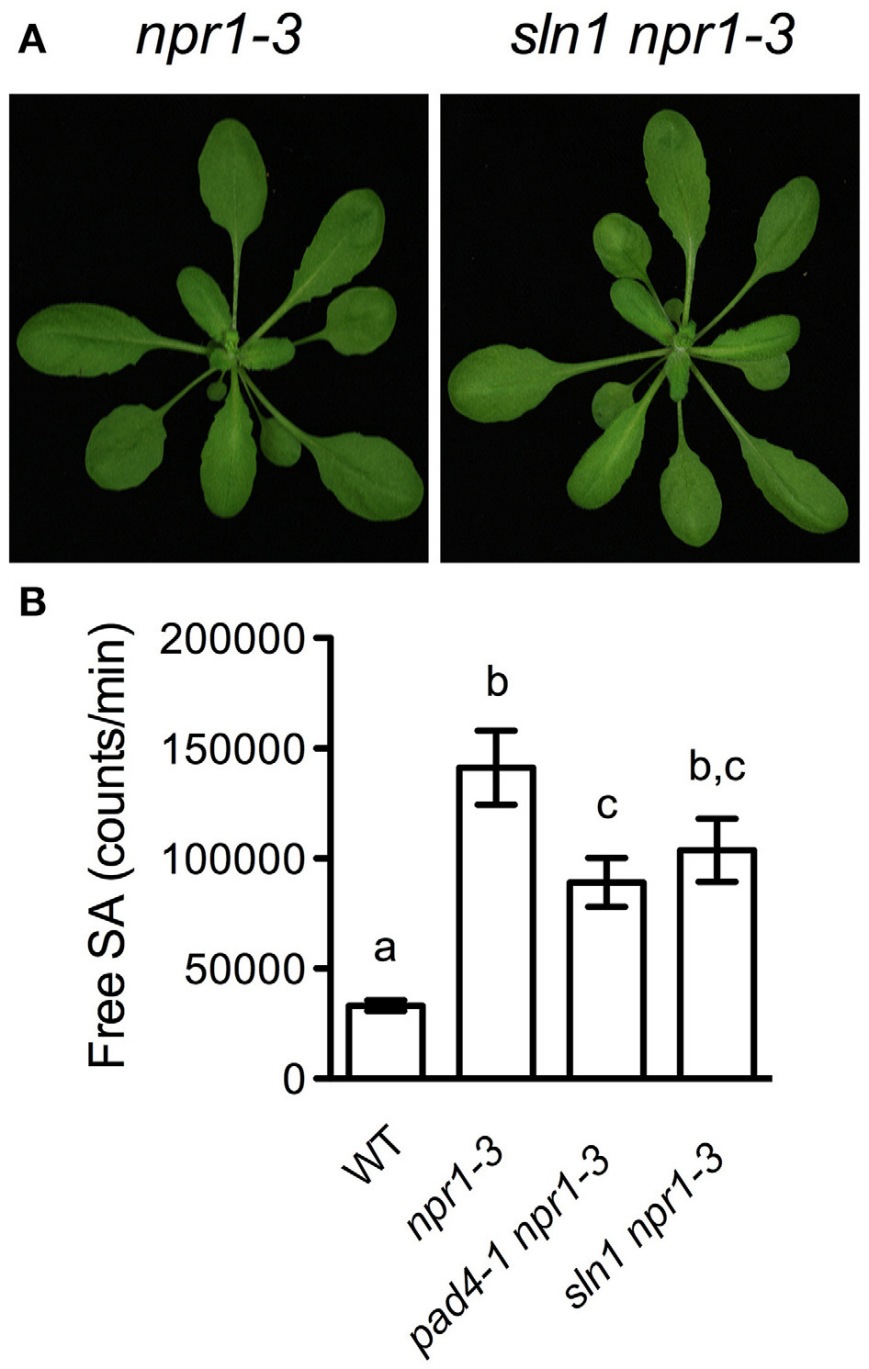
**Further characterization of *sln1 npr1-3*. (A)** Photos of 4-week-old soil-grown *npr1-3* and *sln1 npr1-3* plants. **(B)** Luminescence from crude extracts of *Pst* DC3000/*avrRpt2*-infected wild-type, *npr1-3*, *pad4-1 npr1-3*, *sln1 npr1-3* leaf tissues measured with the SA biosensor. Values are the mean of eight independent samples with SD. Different letters above the bars indicate significant differences (*P* < 0.05, One-Way ANOVA). The experiment was repeated three times with similar results.

To have a better understanding of the *sln* mutations, we further characterized one of the newly identified SA accumulation mutants, *sln1 npr1-3*. The *sln1 npr1-3* mutant was morphologically similar to *npr1-3* (Figure 3A). F_1_ plants from a backcross of *sln1 npr1-3* and *npr1-3* accumulated similar levels of free SA as *npr1-3*, suggesting that *sln1* is recessive. SA analysis of F_2_ progeny showed that *sln1* segregated as a single Mendelian locus (high SA:low SA, 33:8; χ^2^ = 0.6585, 0.25 < P < 1).

It was reported that the *pad4* mutation does not affect free SA accumulation in response to the avirulent bacterial pathogen *Pst* DC3000/*avrRpt2* (Zhou et al., 1998). To test whether the *sln1* mutation influences the avirulent pathogen-induced SA accumulation, we challenged *sln1 npr1-3* plants with *Pst* DC3000/*avrRpt2*. As shown in Figure 3B, *Pst* DC3000/*avrRpt2* induced significant SA accumulation in both *sln1 npr1-3* and *pad4-1 npr1-3* plants. Although free SA levels accumulated in the *sln1 npr1-3* plants were still slightly lower than those in the *npr1-3* plants, the difference was not as dramatic as that detected in the *Psm* ES4326-infected plants (Figure 1). These results indicate that the avirulent pathogen *Pst* DC3000/*avrRpt2*-triggered SA accumulation is largely independent of *SLN1*.

### SA accumulation in the *sln1* single mutant

**Figure 4:**
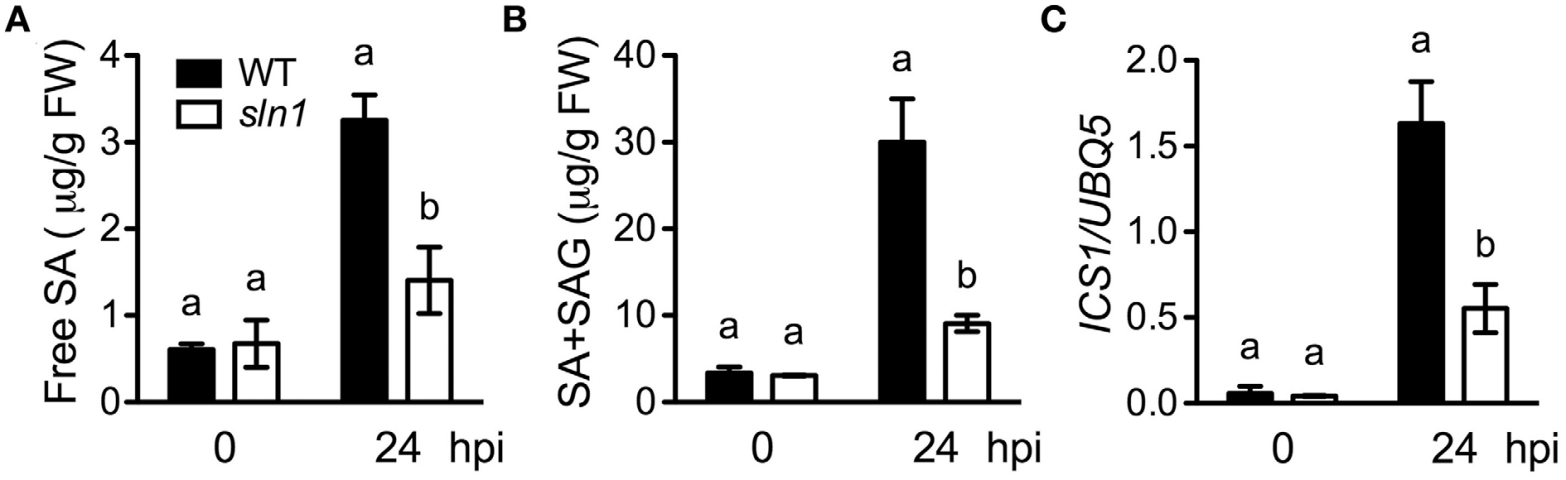
**Pathogen-induced SA levels and *ICS1* expression in the *sln1* single mutant**. Leaves of 4-week-old soil-grown wild-type and *sln1* plants were infiltrated with a suspension of *Psm* ES4326 (OD_600_ = 0. 001). The inoculated leaves were harvested 24 h post-inoculation (hpi) for SA measurement using HPLC or *ICS1* expression analysis using qPCR. **(A)** Free SA levels in *Psm* ES4326-infected wild-type and *sln1* plants. **(B)** Total SA levels in *Psm* ES4326-infected wild-type and *sln1* plants. **(C)** *ICS1* expression levels in *Psm* ES4326-infected wild-type and *sln1* plants. Values are the mean of three independent samples with SD. Different letters above the bars indicate significant differences (*P* < 0.05, Student's *t*-test). The comparison was made separately for each time point. Expression of *ICS1* in **(C)** was normalized against constitutively expressed *UBQ5*. The experiments were repeated three times with similar results.

Since the *sln1* mutation is able to reduce SA accumulation in *npr1-3*, it may affect SA accumulation in the presence of *NPR1*. To test this, we isolated *sln1* single mutant in the F_2_ progeny of a cross between *sln1 npr1-3* and the wild-type Col-0 using the *npr1-3* dCAPS marker (Table S1) and based on SA levels accumulated in the plants upon *Psm* ES4326 infection. As shown in Figures 4A,B, both free SA and total SA levels accumulated in the *sln1* single mutant plants after *Psm* ES4326 infection were significantly lower than those in the wild type. We also found that *Psm* ES4326-induced expression of *ICS1*, which is responsible for pathogen-induced SA accumulation (Wildermuth et al., 2001), was significantly reduced in the *sln1* single mutant compared with that in the wild type (Figure 4C), indicating that *SLN1* may regulate SA accumulation through *ICS1*.

### Pathogen resistance of the *sln1* single mutant

**Figure 5:**
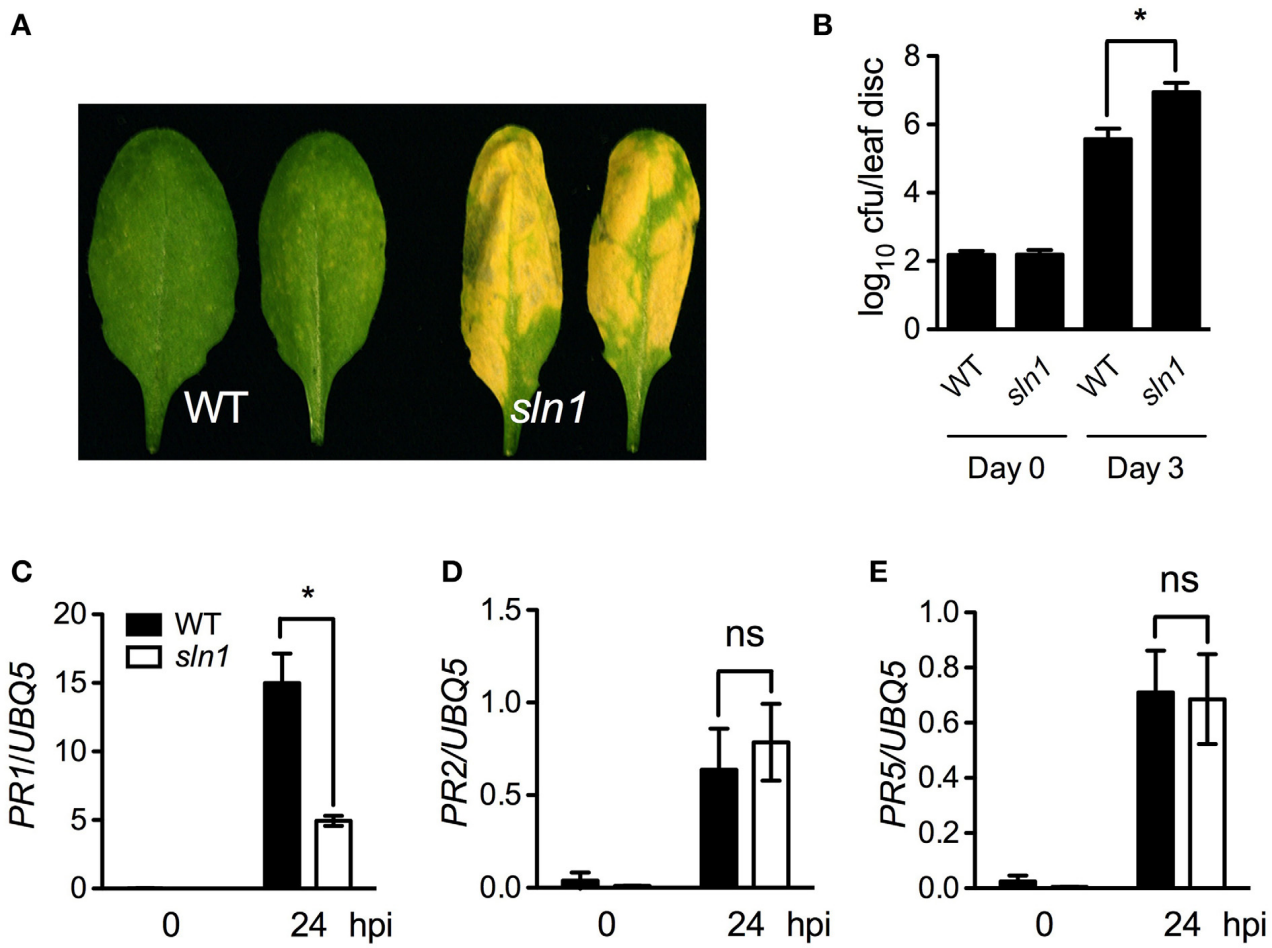
**Defense responses in the *sln1* single mutant. (A)** Disease symptoms of *Psm* ES4326-infected wild-type and *sln1* leaves. Four-week-old soil-grown plants were inoculated with a suspension of *Psm* ES4326 (OD_600_ = 0.0001). Photos were taken 3 days post-inoculation. **(B)** Growth of *Psm* ES4326 in wild-type and *sln1* plants. Four-week-old soil-grown plants were inoculated with a suspension of *Psm* ES4326 (OD_600_ = 0.0001). The *in planta* bacterial titers were determined immediately and 3 days post-inoculation. Data represent the mean of eight independent samples with SD. **(C–E)** *Psm* ES4326-induced *PR1* **(C)**, *PR2* **(D)**, and *PR5* **(E)** gene expression in wild-type and *sln1* plants. Four-week-old soil-grown plants were inoculated with a suspension of *Psm* ES4326 (OD_600_ = 0.001). Total RNA was extracted from leaf tissues collected at 24 hpi and subjected to qPCR analysis. Data represent the mean of three independent samples with SD. An asterisk (^*^) above the bars indicates significant differences (*P* < 0.05, Student's *t*-test). ns, not significant. All experiments were repeated three times with similar results.

We then investigated pathogen growth in the *sln1* single mutant. After infected with a low-titer inoculum (OD_600_ = 0.0001) of *Psm* ES4326, the *sln1* single mutant plants developed enhanced disease symptoms (Figure 5A), and supported ~15-fold more bacterial growth than the wild type (Figure 5B). We also tested pathogen-induced *PR* gene expression in the *sln1* single mutant. As shown in Figures 5C–E, *Psm* ES4326-induced *PR1* expression was significantly reduced in the *sln1* single mutant, but the induction of *PR2* and *PR5* in *sln1* was comparable to that in the wild type. Taken together, these results indicate that *SLN1* is required for defense responses against the bacterial pathogen *Psm* ES4326.

**Figure 6:**
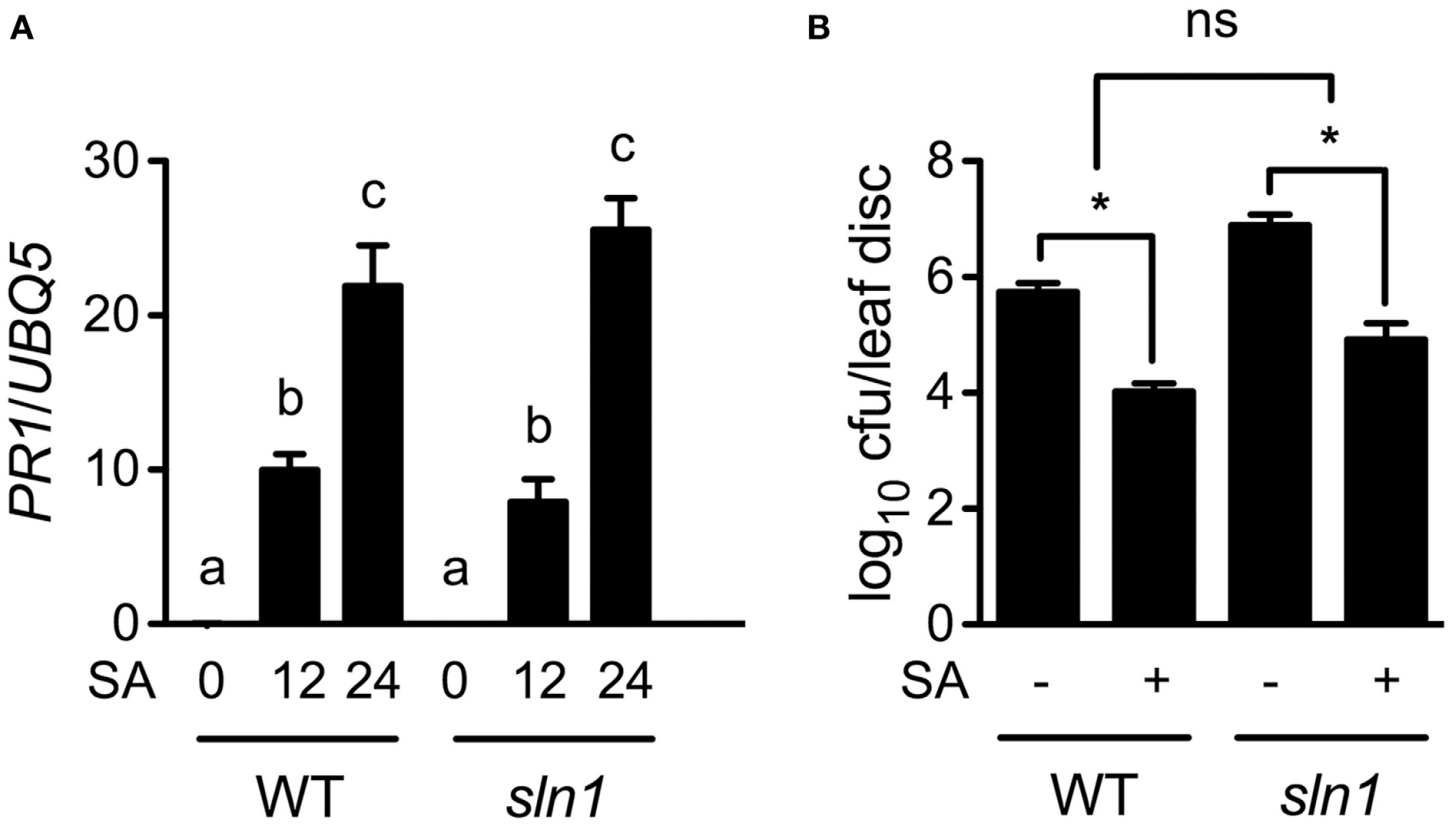
**Exogenous SA-induced *PR* gene expression and resistance in *sln1*. (A)** Exogenous SA-induced *PR1* expression in wild-type and *sln1* plants. Four-week-old soil-grown wild-type and *sln1* plants were soaked with an SA water solution (1 mM). Total RNA was extracted from leaf tissues collected at the indicated time points and analyzed for *PR1* expression using qPCR. Values are the mean of three independent samples with SD. Different letters above the bars indicate significant differences (*P* < 0.05, One-Way ANOVA). The comparison was made separately for each genotype. **(B)** Exogenous SA-induced resistance to *Psm* ES4326 in wild-type and *sln1* plants. Plants were treated as in **(A)**. Twelve hours later, the plants were inoculated with a suspension of *Psm* ES4326 (OD_600_ = 0.001). The *in planta* bacterial titers were determined 3 days post-inoculation. Values are the mean of eight independent samples with SD. An asterisk (^*^) above the bars indicates significant differences (*P* < 0.05, Two-Way ANOVA). ns, not significant. These experiments were repeated three times with similar results.

Since the *sln1* mutation inhibits pathogen-induced SA accumulation, exogenous SA may restore defense responses in *sln1* plants. Indeed, SA treatment induced similar levels of *PR1* gene expression and resistance to *Psm* ES4326 in the *sln1* single mutant and the wild-type plants (Figures 6A,B). Based on these results, we concluded that the signaling pathway downstream of SA in *sln1* is intact. Thus, *SLN1* most likely functions in a signal amplification loop upstream of SA.

### Preliminary mapping of the *sln1* mutation

**Figure 7:**
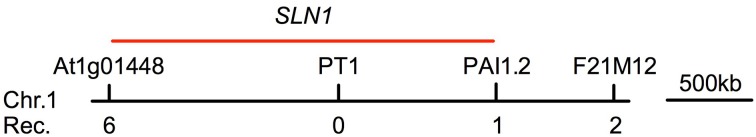
**Preliminary mapping of the *sln1* mutation**. A total of 74 F_2_ progeny homozygous for *sln1* were used to determine the approximate position of the *sln1* mutation using bulked segregant analysis. The *sln1* mutation was linked to the molecular marker PT1 on chromosome 1. Out of the 74 F_2_ plants, six were heterozygous at gene At1g01448, and one was heterozygous at the molecular marker PAI1.2. The heterozygotes found by these two markers were mutually exclusive. No heterozygotes were found at PT1. The *SLN1* gene is likely located in the vicinity of PT1, as indicated by the red bar. Rec., recombinant.

To map the *sln1* mutation, *sln1 npr1-3* (in the Col-0 genetic background) was crossed with *npr1*-L (an *npr1* T-DNA insertion mutant in the polymorphic ecotype Landsberg *erecta*) to generate a segregating population. Preliminary mapping using 74 F_2_ plants, which accumulated extremely low levels of SA after *Psm* ES4326 infection, revealed that *sln1* is located between gene At1g01448 and the molecular marker PAI1.2 (Figure 7). To our knowledge, this region does not contain any known genes regulating pathogen-induced SA accumulation. Therefore, *SLN1* likely encodes a new regulator of SA biosynthesis. Further fine-mapping and/or whole genome sequencing will help identify the *sln1* mutation.

## Discussion

In this study, we performed a forward genetic screen for Arabidopsis mutants with altered SA accumulation during pathogen infection using the newly developed SA biosensor method (Marek et al., 2010). Compared with the HPLC and GC/MS approaches, the SA biosensor method is much faster and less expensive (Malamy et al., 1992; Verberne et al., 2002; Marek et al., 2010). Using this method, we screened a large population (35,000) of M_2_ plants in less than 1 year. Approximately 350 putative SA accumulation mutants in the *npr1-3* genetic background were identified. Among them, 17 are *sln npr1-3* mutants, producing significantly lower levels of SA than *npr1-3* after *Psm* ES4326 infection, and two are *isn npr1-3* mutants, accumulating higher levels of SA than *npr1-3* (Figures 1A,B). Interestingly, upon *Psm* ES4326 infection, SA levels accumulated in the remaining putative mutants (in the *npr1-3* background) were significantly lower than those in *npr1-3*, but slightly higher than those in the wild-type plants, suggesting the existence of a larger number of regulatory components involved in pathogen-induced SA accumulation. Indeed, genetic studies have uncovered a complicated signaling network that regulates SA accumulation. This consists of upstream SA signaling components (such as EDS1, PAD4, and NDR1), downstream SA signaling components (such as NPR1), transcription factors (such as CBP60g and SARD1), metabolic enzymes (such as EPS1 and PBS3), and various positive and negative feedback loops (Cao et al., 1997; Ryals et al., 1997; Shah et al., 1997; Zhou et al., 1998; Jirage et al., 1999; Shapiro and Zhang, 2001; Wildermuth et al., 2001; Jagadeeswaran et al., 2007; Lee et al., 2007; Nobuta et al., 2007; Zheng et al., 2009; Zhang et al., 2010; Wang et al., 2011).

The SA accumulation phenotype of the *sln* mutants is similar to that of *eds5*, *sid2*, and *pad4* mutants (Zhou et al., 1998; Jirage et al., 1999; Nawrath and Métraux, 1999; Wildermuth et al., 2001; Nawrath et al., 2002). *EDS5* and *SID2* encode a chloroplast MATE transporter and an SA biosynthetic enzyme ICS1, respectively, which are two important components in the SA biosynthesis pathway. PAD4 is a lipase-like protein involved in an SA positive signal-amplification loop required for activation of defense responses (Jirage et al., 1999). Complementation tests indicated that seven out of the 17 *sln* mutants are new alleles of *eds5*, two are alleles of *sid2*, and one is allelic to *pad4*, and the other seven *sln* and two *isn* mutants are new non-allelic mutants (Table 1). Although this is a large-scale genetic screen, the low frequency of alleles for the new *sln* and *isn* mutants indicates that our genetic screen has not been saturated.

Several other recessive mutations have also been reported to impair pathogen-induced SA accumulation. In the *eps1-1* mutant, pathogen-induced accumulation of SAG was greatly reduced, but free SA levels were comparable to those in the wild type. *EPS1* encodes a novel member of the BAHD acyltransferase superfamily, which is predicted to be directly involved in the synthesis of a precursor or regulatory molecule for SA biosynthesis (Zheng et al., 2009). Similarly, the *pbs3-1* mutant displayed decreased pathogen-induced accumulation of SAG, but varied in free SA accumulation between studies (Jagadeeswaran et al., 2007; Lee et al., 2007; Nobuta et al., 2007). PBS3 belongs to the acyl adenylate/thioesterforming enzyme superfamily. The exact functions of both EPS1 and PBS3 in SA biosynthesis, however, have not been clearly defined. Consistent with these studies, we found that free SA levels in the *eps1-1* and *pbs3-1* mutants were comparable to those in the wild type when assayed with the SA biosensor (Figure S1). Thus, the *sln* mutations are unlikely located in either *EPS1* or *PBS3*, since these mutations greatly influenced *Psm* ES4326-induced free SA accumulation (Figures 1A,B). Additionally, although the *eds1* mutation significantly affects pathogen-induced accumulation of both free SA and SAG (Falk et al., 1999), the *sln* mutants are unlikely alleles of *eds1*, because there are two *EDS1* genes lying in tandem on chromosome 3 of the Arabidopsis ecotype Col-0 (Feys et al., 2005). Therefore, the *SLN* genes may encode new signaling components downstream of recognition of pathogen infection, or new enzymes directly involved in the synthesis of a precursor and/or regulatory molecule for SA biosynthesis.

In addition to components upstream of SA biosynthesis, the downstream component, NPR1 (nonexpressor of *PR* genes1), which has been shown to be an important regulator of defense responses (Cao et al., 1997; Dong, 2004), also regulates SA levels. Mutations in the *NPR1* gene enhance SA accumulation during pathogen infection, suggesting that NPR1 is a feedback inhibitor of SA biosynthesis (Figures 1A,B; Clarke et al., 2000; Wildermuth et al., 2001; Zhang et al., 2010). Here we found that *eds5 npr1-3*, *sid2 npr1-3*, *pad4 npr1-3*, and *sln npr1-3* double mutants accumulated significantly lower levels of SA than *npr1-3* (Figures 1A,B), suggesting that these mutations (*eds5*, *sid2*, *pad4*, and *sln*) suppress *npr1*-mediated SA hyperaccumulation. On the other hand, these double mutants were more susceptible to *Psm* ES4326 than *npr1-3* (Figure 2), indicating that *EDS5*, *SID2*, *PAD4*, and the *SLN* genes may contribute to NPR1-independent defense responses (Glazebrook, 2001). NPR1-independent defense signaling pathways have been shown to be activated in various Arabidopsis mutants, including *sni1* (Li et al., 1999), *snc1* (Li et al., 2001), *ssi* (Shah et al., 1999, 2001), and *cpr* (Bowling et al., 1997; Clarke et al., 1998). The two *isn* mutations appear to also activate NPR1-independent disease resistance (Figure 2).

We further isolated and characterized the *sln1* single mutant. The *sln1* plants exhibited significantly reduced levels of *Psm* ES4326-induced SA and supported more *Psm* ES4326 growth than the wild-type plants (Figures 4A,B, 5B), suggesting that *SLN1* plays an important role in activation of defense responses against this pathogen. Interestingly, the *sln1* mutation appears to differentially influence pathogen-induced *PR1*, *PR2*, and *PR5* expression. *Psm* ES4326-induced *PR1* expression was greatly reduced in *sln1* plants, but induction of *PR2* and *PR5* was nearly unaffected (Figures 5C–E). In this regard, *sln1* is also similar to *eds5*, *sid2*, and *pad4*, which cause reduced induction of *PR1*, but have no effect on the expression of *PR2* and *PR5* (Rogers and Ausubel, 1997; Zhou et al., 1998; Nawrath and Métraux, 1999). On the other hand, pathogen-induced expression of *PR1*, *PR2*, and *PR5* is strongly reduced in *NahG* transgenic plants (Nawrath and Métraux, 1999), which argues against the idea that an SA-independent pathway exists to control *PR2* and *PR5* expression. It is possible that the low levels of SA accumulated in the SA biosynthesis mutants are sufficient for induction of *PR2* and *PR5*, but not for *PR1*.

In summary, we identified a group of new SA accumulation mutants, including seven *sln* mutants and two *isn* mutants, in a genetic screen using the newly developed SA biosensor-based method. Further characterization of these *sln* and *isn* mutants and cloning of the *SLN* and *ISN* genes will shed new light on the molecular mechanisms underlying pathogen-induced SA accumulation and SA-mediated defense signaling in plants.

## Author contributions

Yezhang Ding and Zhonglin Mou designed research. Yezhang Ding and Danjela Shaholli performed mutant screen. Yezhang Ding characterized mutants and analyzed data. Yezhang Ding and Zhonglin Mou wrote the manuscript.

### Conflict of interest statement

The authors declare that the research was conducted in the absence of any commercial or financial relationships that could be construed as a potential conflict of interest.

## Supplementary material

Figure S1 **SA accumulation in *eps1* and *pbs3***. Luminescence from crude extracts of *Psm* ES4326-infected wild-type, *npr1-3*, *eps1-1*, and *pbs3-1* leaf tissues measured with the SA biosensor. Values are the mean of six samples with standard deviation (SD). The experiment was repeated with similar results.
