# Supplementary material for: A large-scale genetic screen for mutants with altered salicylic acid accumulation in Arabidopsis
Source: Front Plant Sci. 2015 Jan 7;5:763. doi: 10.3389/fpls.2014.00763 (PMC4285869; doi:10.3389/fpls.2014.00763)
Supplement: Supplementary file 1 [file DataSheet1.DOCX]

**Table S1.** The CAPS and dCAPS markers used in this study

| Marker | Forward primer (5’-3’) | Reverse primer (5’-3’) | Restriction enzyme | WT (bp) | Mutant  (bp) |
| --- | --- | --- | --- | --- | --- |
| *npr1-3*  (dCAPS) | GGCCGACTATGTGTAGAAATACTAGCG | TGAGACGGTCAGGCTCGAGG | HhaI | 319, 27 | 346 |
| *eds5-1* | CTTGGTCTAATCTGATTCTTGATATGTTTGCCG | GAGACTTATTCAGCTGCTTGCTTCTC | HpaII | 142, 29 | 171 |
| *pad4-1* | CTGGTTCTGTTCGTGGATG | ATCTGCTTCTCACACACTCC | BSMF1 | 468,  234 | 702 |
| At1g01448 | CCCAAAGCTATACACGTCAG | GAGAATATACCACGGAGAG | Taq I | 267 | 236, 31 |
| *sid2-1* | AAGCTTGCAAGAGTGCAA | AATTAATCGCCTGTAGAGATGTTG |  | 800 | nothing |

**Table S2.** Primers used for qPCR in this study

| Gene | Forward primer (5’-3’) | Reverse primer (5’-3’) |
| --- | --- | --- |
| *PR1* | CTCATACACTCTGGTGGG | ATTGCACGTGTTCGCAGC |
| *PR2* | ATCAAGGAGCTTAGCCTCAC | TGTAAAGAGCCACAACGTCC |
| *PR5* | CTCTTCCTCGTGTTCATCAC | GAAGCACCTGGAGTCAATTC |
| *ICS1* | GAATTTGCAGTCGGGATCAG | AATTAATCGCCTGTAGAGATGTTG |
| *UBQ5* | TCTCCGTGGTGGTGCTAAG | GAACCTTTCCAGATCCATCG |
