# Supplementary figures and images for: A large-scale genetic screen for mutants with altered salicylic acid accumulation in Arabidopsis
Source: Front Plant Sci. 2015 Jan 7;5:763. doi: 10.3389/fpls.2014.00763 (PMC4285869; doi:10.3389/fpls.2014.00763)

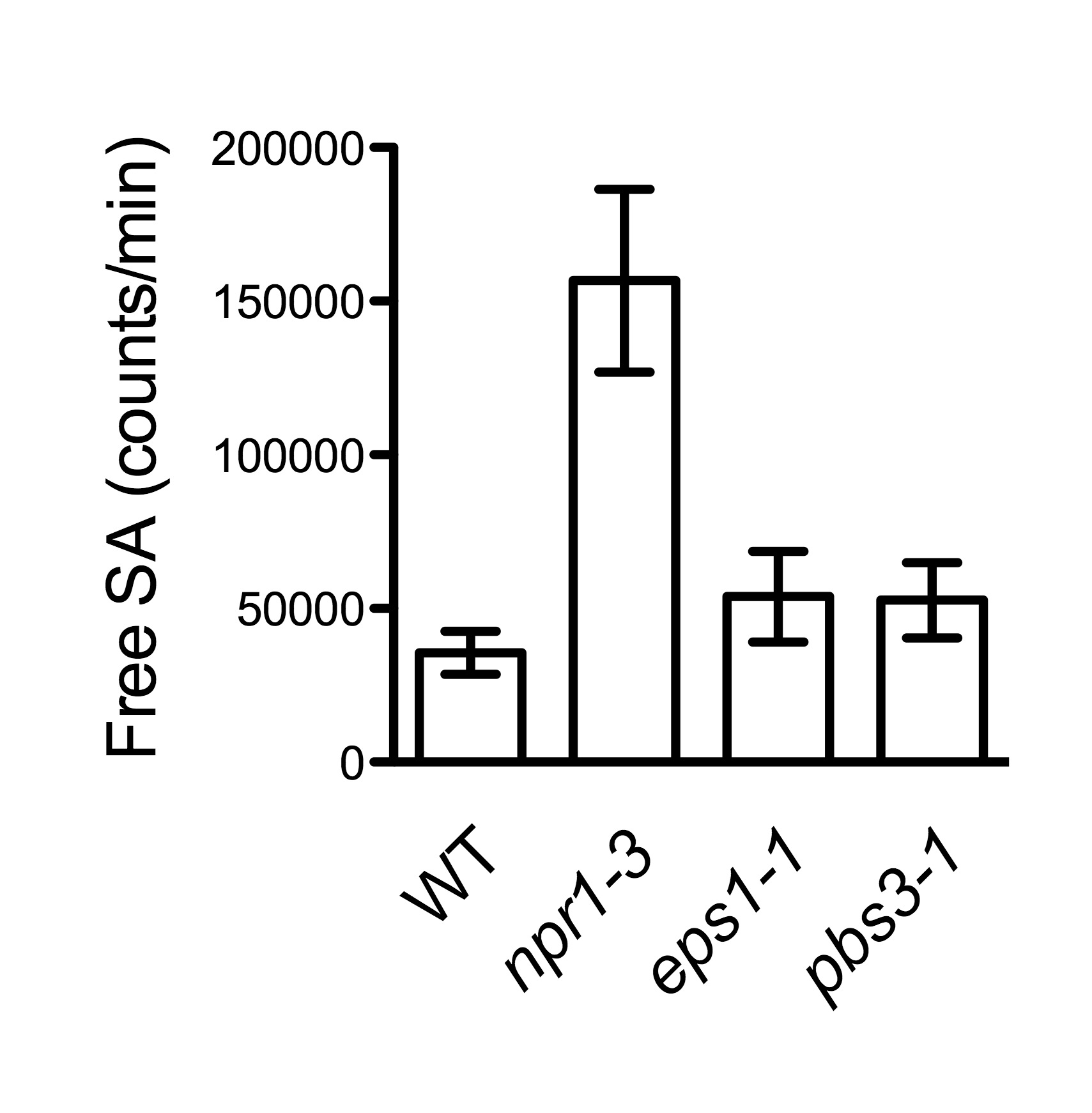

Supplement: Figure S1 — SA accumulation in eps1 and pbs3. Luminescence from crude extracts of Psm ES4326-infected wild-type, npr1-3, eps1-1, and pbs3-1 leaf tissues measured with the SA biosensor. Values are the mean of six samples with standard deviation (SD). The experiment was repeated with similar results. [file Image1.JPEG]
